# Association of neutrophil-to-lymphocyte ratio, radiotherapy fractionation/technique, and risk of development of distant metastasis among patients with locally advanced rectal cancer

**DOI:** 10.1186/s13014-022-02065-8

**Published:** 2022-05-21

**Authors:** Gowoon Yang, Jee Suk Chang, Jeong Eun Choi, Eun Sil Baek, Seung-Seob Kim, Hwa Kyung Byun, Yeona Cho, Woong Sub Koom, Seung Yoon Yang, Byung Soh Min, Sang Joon Shin

**Affiliations:** 1 Department of Radiation Oncology, Yonsei Cancer Center, Yonsei University College of Medicine, Seoul, Republic of Korea; 2 Department of Radiation Oncology, Gangnam Severance Hospital, Yonsei University College of Medicine, Seoul, Republic of Korea; 3 Songdang Institute for Cancer Research, Yonsei University College of Medicine, Seoul, Republic of Korea; 4 Department of Radiology, Severance Hospital, Yonsei University College of Medicine, Seoul, Republic of Korea; 5 Department of Surgery, Yonsei Cancer Center, Yonsei University College of Medicine, Seoul, Republic of Korea; 6 Department of Internal Medicine, Yonsei Cancer Center, Yonsei University College of Medicine, Seoul, Republic of Korea

**Keywords:** Neutrophil, Lymphocyte, Distant metastasis-free survival, Poor outcome, Rectal neoplasm

## Abstract

**Background:**

We investigated the prognostic impact of the neutrophil-to-lymphocyte ratio (NLR) in patients with locally advanced rectal cancer (LARC) and whether modifiable factors in radiotherapy (RT) influenced the NLR.

**Methods:**

Data of 1386 patients who were treated with neoadjuvant RT and concurrent or sequential chemotherapy for LARC between 2006 and 2019 were evaluated. Most patients (97.8%) were treated with long-course RT (LCRT; 50–50.4 Gy in 25–28 fractions) using three-dimensional conformal radiotherapy (3D-CRT) (n = 851) or helical tomotherapy (n = 504), and 30 patients underwent short-course RT (SCRT; 25 Gy in 5 fractions, followed by XELOX administration for 6 weeks). Absolute neutrophil and lymphocyte counts were obtained at initial diagnosis, before and during the preoperative RT course, and after preoperative concurrent chemoradiotherapy. The primary endpoint was distant metastasis-free survival (DMFS).

**Results:**

The median follow-up time was 61.3 (4.1–173.7) months; the 5-year DMFS was 80.1% and was significantly associated with the NLR after RT but not before. A post-RT NLR ≥ 4 independently correlated with worse DMFS (hazard ratio, 1.42; 95% confidence interval, 1.12–1.80), along with higher ypT and ypN stages. Post-RT NLR (≥ 4) more frequently increased following LCRT (vs. SCRT, odds ratio [OR] 2.77, *p* = 0.012) or helical tomotherapy (vs. 3D-CRT, OR 1.29, *p* < 0.001).

**Conclusions:**

Increased NLR after neoadjuvant RT is associated with increased distant metastasis risk and poor survival outcome in patients with LARC. Moreover, high NLR following RT is directly related to RT fractionation, delivery modality, and tumor characteristics. These results are hypothesis-generating only, and confirmatory studies are required.

**Supplementary Information:**

The online version contains supplementary material available at 10.1186/s13014-022-02065-8.

## Background

Though a combination of neoadjuvant chemoradiotherapy and total mesorectal excision (TME) decreases the local recurrence rate to < 5–10% in patients with locally advanced rectal cancer (LARC), distant metastasis (DM) is currently the main cause of treatment failure (25–40%) [1]. Evidence supports both long-course and short-course radiotherapy (LCRT and SCRT) for patients with LARC; however, SCRT has not yet been widely adopted globally [2]. Though recent studies have focused on total neoadjuvant therapy (TNT), which delivers chemotherapy and radiation before TME, overtreatment and lack of identification of patients with more aggressive diseases are clinically relevant issues [3].

The ability of tumors to invade and result in metastases is dependent on the intrinsic characteristics of both tumor cells and the environment around the tumor, i.e., host factors, including anti-cancer immunity. In recent years, there has been growing interest in systemic inflammatory responses, and the neutrophil-to-lymphocyte ratio (NLR) has been suggested as a simple index to study in patients critically ill and with cancer. In previous studies, increased NLR was associated with decreased overall survival in various cancer types [4, 5]. Furthermore, owing to the exquisite radiosensitivity of circulating lymphocytes, which is required for the anti-tumoral immune response, RT is often accompanied by lymphopenia, which can subsequently influence not only NLR but also cancer recurrence and survival [6].

As only few studies have focused on the prediction of DM using NLR in patients with LARC, we aimed to investigate whether NLR in the peripheral blood of patients with LARC correlated with DM-free survival (DMFS) and whether RT modalities could affect changes in NLR. The findings of our study could help in identifying high risk groups that may benefit from the intensification of preoperative therapy, including TNT, and confirm whether the modification of RT modalities could result in improved outcomes for these patients by better addressing NLR changes.

## Methods

### Patients

The Institutional Review Board of Yonsei Cancer Center reviewed and approved the study protocol (IRB Number 4-2021-0318). The requirement for informed consent was waived owing to the retrospective nature of this study. Data regarding tumor stage, pathology, number of total lymph nodes examined, positive lymph nodes, and survival outcomes were obtained from electronic medical records.

Patients were required to have a biopsy-proven diagnosis of rectal cancer along with clinical, radiographic, and pathologic assessments of LARC for staging purposes. All the patients in the present study had undergone rectal magnetic resonance imaging (MRI) for initial staging. The tumor-node-metastasis (TNM) staging was performed according to the MRI findings, which were read by a radiologist. The rectal tumors were classified into four categories (a–d) according to the extent of extramural spread: < 1 mm, 1–5 mm, > 5–15 mm, and > 15 mm, as proposed by Compton and Greene in 2004 [7]. A lymph node was considered suspicious if the short axis of the lymph node was longer than 5 mm or if the margin of the lymph node was irregular or showed heterogenous signal intensity. Patients presenting with stage IV rectal cancer at initial diagnosis were excluded from the analysis. Patients with a histology other than an adenocarcinoma or those who did not undergo surgery after concurrent chemoradiotherapy (CCRT) were also excluded. None of the patients underwent total neoadjuvant treatment in this study.

A cohort of 1386 patients who were diagnosed with rectal cancer and treated with preoperative CCRT between 2006 and 2019 were included in this study. Among them, those who presented with DM at initial diagnosis (n = 22), had a histology other than an adenocarcinoma (n = 5), or who did not undergo surgery after CCRT (n = 4) were excluded. Thus, a total of 1355 patients were included for analysis. Adjuvant chemotherapy was administered when the patient was pathologically node-positive. In pathologically node-negative patients, those with ypT3-4 tumors or other high-risk features received adjuvant chemotherapy.

### Statistical analyses

Absolute neutrophil and lymphocyte counts were obtained from the sample collected at initial rectal cancer diagnosis, before and during the preoperative CCRT course, and within 3 months after preoperative CCRT. NLR was calculated by dividing the absolute neutrophil count with the lymphocyte count.

The primary endpoint was DMFS. To determine the NLR cut-off value, a univariate analysis of DMFS with repeated Cox regression models was conducted for each NLR value in 0.5–1.0 increments, as per our statistical team suggestion. The corresponding hazard ratio (HR), 95% confidence interval (CI), and *p* value for each NLR value were obtained. The NLR cut-off value with the lowest *p* value and highest HR was selected as the optimal value. The patient cohort was split into two groups according to the optimal discriminative NLR cut-off value. In addition, to estimate the probability of NLR reaching its peak, the Kaplan–Meier curve was drawn. The peak NLR value was defined as the maximum value of NLR evaluated from the diagnosis of rectal cancer till surgery.

Other endpoints included overall survival (OS), local recurrence-free survival (LRFS), pathologic complete response (pCR), and neoadjuvant rectal score (NAR). OS was defined as the time from initial diagnosis to all-cause death, DMFS was defined as the time from initial diagnosis to the occurrence of DM at any site, and disease-free survival (DFS) was defined as the time from initial diagnosis to cancer recurrence or all-cause death. These three factors are expressed in months. The Kaplan–Meier curve for 5-year OS and DMFS was obtained. The NAR score was calculated based on the clinical T stage and pathologic T and N stages [8]. This score is a continuous variable that ranges from 0 to 100, with higher scores representing poorer prognosis. The NAR score was designed to represent changes in factors that are affected by neoadjuvant therapy. Student’s t-test was used to analyze the association between NLR and NAR scores.

All analyses were performed using IBM SPSS Statistics version 25.0 for Windows (IBM Corp. Armonk, NY, USA). Each continuous variable was manually reviewed to identify outliers, and extreme values were double checked in the data source for possible correction; these values were otherwise excluded from the analysis. After basic data cleaning, patient demographics and tumor characteristic data were analyzed. Data are presented as means, medians, standard deviations (SDs), ranges, and percentages.

Student’s t-test was used for continuous variables, and the chi-square test was used for categorical variables to compare differences between both groups. A two-sided *p* value < 0.05 indicated statistical significance. Univariate and multivariate survival analyses were conducted using Cox proportional hazard models. The obtained models were used to analyze the association between survival outcomes and variables, including NLR, age at diagnosis, sex, initial T stage, node metastatic status, year of diagnosis, RT modality and fractionation, T stage after CCRT, the chemotherapy regimen used for CCRT, and whether adjuvant chemotherapy was administered following surgery.

## Results

The mean age of patients was 59.7 years (SD, 12 years), and 64.9% of patients were men. A total of 1009 patients presented with tumor stage T3 at initial diagnosis (74.5%), 202 with T4 (14.9%), and 131 with Tis-T2 (9.7%). Moreover, 1049 patients (77.4%) presented with node metastases at initial diagnosis. Regarding the tumor location, 675 patients had their tumor located in the mid-rectum (49.8%) and 559 patients had their tumor located in the lower rectum (41.3%). A total of 794 patients were treated with capecitabine-based CCRT (58.6%), and 422 patients (31.1%) were treated with fluorouracil.

Adjuvant chemotherapy was performed for 53.7% of patients postoperatively (n = 727). As for the RT modality, 851 patients were treated with 3D-CRT (62.8%) and 504 patients were treated with tomotherapy (37.2%). Thirty patients were treated with SCRT (2.2%, 25 Gy in 5 fractions), and the rest of the patients were treated with conventional fractionation (LCRT, 50–50.4 Gy in 25–28 fractions). All patients treated with SCRT received 1-week SCRT plus 6-week XELOX (capecitabine 1000 mg/m^2^) and oxaliplatin 130 mg/m^2^ every 3 weeks before TME.

The corresponding details are summarized in Table 1.

**Table 1:** Demographic and clinical characteristics of the patients (N = 1355)

Variables	N	%
Age, years		
Mean (SD)	59.7	SD 12.0
< 60	667	49.2
≥ 60	688	50.8
Sex		
Female	475	35.1
Male	880	64.9
Clinical T stage		
Tis-T2	131	9.7
T3/4	1211	89.4
Tx	13	1.0
Clinical N stage		
N0	252	18.6
N1/2	1049	77.4
Nx	54	4.0
Distance of tumor from the anal verge		
Lower	559	41.3
Middle	675	49.8
Upper	121	8.9
Year of diagnosis*		
< 2013	511	37.7
≥ 2013	844	62.3
ypT		
Tis/0	286	21.1
T1/2	381	28.1
T3/4	682	50.3
Tx	6	0.4
ypN		
N0	980	72.3
N1/2	372	27.5
Nx	3	0.2
Neoadjuvant chemotherapy		
Capecitabine	794	58.6
FL	422	31.1
Others	139	10.3
Adjuvant chemotherapy		
FL	277	20.4
Capecitabine	230	17.0
FOLFOX	204	15.1
Others	16	1.2
None	628	46.3
RT modality		
3D-CRT	851	62.8
Tomotherapy	504	37.2
RT fractionation		
LCRT	1325	97.8
SCRT	30	2.2

*SD* standard deviation, *FL* fluorouracil, *3D-CRT* three-dimensional conformal radiotherapy, *LCRT* long-course RT, *SCRT* short-course RT

*Colorectal cancer multidisciplinary team was implemented in the year 2012–2013 at our institution

**Fig. 1:**
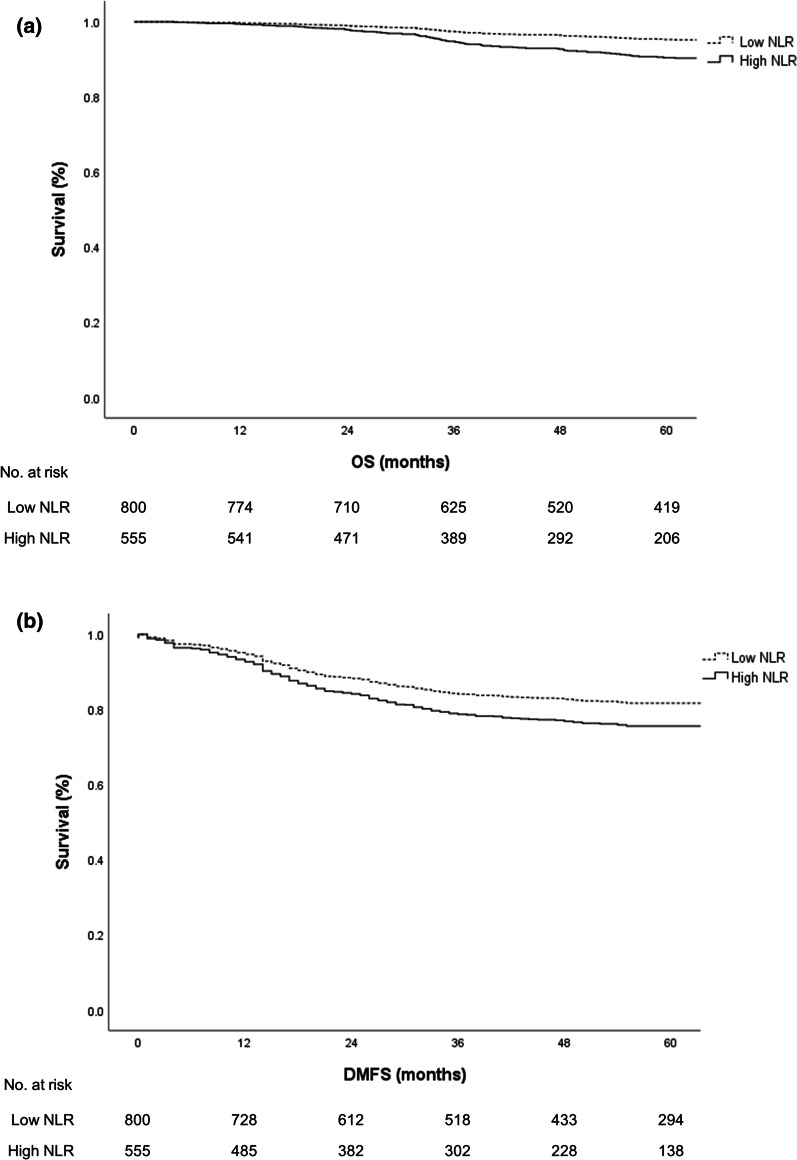
**a** OS in patients with low-post RT NLR versus high post-RT NLR. Cox-adjusted survival curve demonstrating overall survival in patients with low post-radiotherapy (RT) neutrophil-to-lymphocyte ratio (NLR) vs. high post-RT NLR using Cox proportional hazard models (adjusted for age, sex, clinical T stage, clinical N stage, RT modality, ypT, ypN, adjuvant chemotherapy, RT fractionation). **b** DMFS in patients with low-post RT NLR versus high post-RT NLR. Cox-adjusted survival curve demonstrating distant metastasis-free survival in patients with low post-RT neutrophil-to-lymphocyte ratio (NLR) vs. high post-RT NLR using Cox proportional hazard models (adjusted for age, sex, clinical T stage, clinical N stage, RT modality, ypT, ypN, adjuvant chemotherapy, RT fractionation)

The median follow-up time was 61.3 (4.1–173.7) months. During the follow-up period, 58 patients experienced local recurrence and 297 patients presented with DM. NLR data were serially obtained for each patient at initial diagnosis, before and during CCRT, and within 2 months following RT. We defined the NLR obtained after RT completion as post-RT NLR and that obtained before CCRT as pre-RT NLR. There was no significant difference in DMFS between pre- and post-RT NLR values. To determine the cut-off value, we originally conducted the receiver-operating characteristic analysis; however, as there was no statistical significance (area under curve 0.533, *p* > 0.05), it was not used in the present study. Subsequently, we performed statistical analysis with repeated Cox regression models with post-RT NLR values ranging from 3.0 to 7.0 in 1.0 increments, as our statistical team suggested. The post-RT NLR cut-off value which demonstrated the largest difference of DMFS was 4.0 and was selected as the cut-off value in this study (HR = 1.435, 95% CI 1.142–1.803, *p* = 0.002; Additional file 1). A total of 555 patients (41.0%) had a post-RT NLR ≥ 4 and were consequently classified into the high NLR group, whereas the remaining 800 patients (59.0%) had a post-RT NLR < 4 and were consequently classified into the low NLR group. The median post-RT NLR value was 3.1 (range 0.2–17.0, SD 7.1), and the time at which NLR was the highest for each patient was accounted. The post-RT examinations were performed every 2 weeks or 4 weeks after RT was completed. According to the Kaplan–Meier curve, NLR reached its peak at a median of 45 (range 0–90 SD 27.5) days after the initiation of the RT course (Additional file 2). In the short course group, post-RT NLR was evaluated at a median of 35 (range 28–48, SD 7.5) days after evaluation of pre-RT NLR. Post-RT NLR was first evaluated at a median of 9 (range 4–15, SD 2.64) days after RT, and was evaluated repeatedly every 2–4 weeks thereafter. Pre-RT NLR was evaluated at a median of 20 (range 7–43, SD 8.0) days before the RT start date. A high post-RT NLR was associated with poor survival outcomes. The 5-year OS of patients in the high post-RT NLR group was 90.5%, while that of patients in the low post-RT NLR group was 95.3% (*p* < 0.001, Fig. 1a). The 5-year DMFS was 75.6% of patients in the high post-RT NLR group, while that of patients in the low post-RT NLR group was 81.7% (*p* = 0.002, Fig. 1b). However, survival outcomes were not associated with pre-RT NLR. The 5-year OS of patients in the high pre-RT NLR group was 84.2%, while that of patients in the low pre-RT NLR group was 91.8% (*p* = 0.171). The 5-year DMFS of patients in the high pre-RT NLR group was 79.2%, while that of patients in the low pre-RT NLR group was 73.6% (*p* = 0.278). For the multivariate analysis, a Cox proportional hazards model was used. Age, year of diagnosis, post-RT NLR, and post-CCRT tumor T stage were independent factors related to OS (HR = 1.02, *p* < 0.001; HR = 0.51, *p* < 0.001; HR = 2.22, *p* < 0.001; and HR = 2.13, *p* = 0.001, respectively). Age, post-RT NLR and post-CCRT tumor T and N stages were independent factors related to DMFS (HR = 0.99, *p* = 0.017; HR = 1.42, *p* = 0.004; HR = 1.96, *p* < 0.001; and HR = 2.43, *p* < 0.001, respectively, Table 2).

**Table 2:** Multivariate survival analyses for distant metastasis-free survival and overall survival

	DMFS			OS		
	*p* value	HR	95% CI	*p* value	HR	95% CI
Age (≥ 60 vs. < 60 years)	0.017	0.99	0.98–1.00	< 0.001	1.02	1.03–1.06
Sex (male vs. female)	0.606	1.07	0.84–1.36	0.896	0.90	0.60–1.33
Clinical T stage (icT3/4 vs. icT1/2)	0.529	1.20	0.68–2.11	0.479	1.28	0.53–3.08
Clinical N stage (icN1/2 vs. icN0)	0.961	0.93	0.70–1.391	0.160	0.73	0.46–1.14
RT modality (tomotherapy vs. 3D-CRT)	0.459	1.12	0.83–1.53	0.354	0.73	0.38–1.41
Year of diagnosis (> 2013 vs. ≤ 2013)	0.852	0.97	0.72–1.31	< 0.001	0.51	0.51–0.52
PostRT NLR ≥ 4.0 vs. < 4.0	0.004	1.42	1.12–1.80	< 0.001	2.22	1.54–3.20
ypT (ypT3/4 vs. ypT1/2)	< 0.001	1.96	1.46–2.62	0.001	2.13	1.34–3.37
ypN (ypN1/2 vs. ypN0)	< 0.001	2.43	1.80–3.27	0.167	1.37	0.88–2.15
Adjuvant chemotherapy (no vs. yes)	0.552	0.91	0.67–1.24	0.700	0.92	0.60–1.41
RT fractionation (LCRT vs. SCRT)	0.417	0.44	0.06–3.17	0.962	< 0.01	0.00–4.34E+181

*DMFS* distant metastasis-free survival, *OS* overall survival, *HR* hazard ratio, *CI* confidence interval, *NLR* neutrophil-to-lymphocyte ratio, *LCRT* long-course RT, *SCRT* short-course RT

Chi square analysis was performed for pre-RT and post RT NLR. Using intensity-modulated radiation therapy (IMRT) rather than 3D-CRT, and LCRT rather than SCRT, was associated with high post-RT NLR (Table 3).

**Table 3:** Chi square analysis for pre-RT and post-RT NLR

	Pre-RT NLR			Post-RT NLR		
	*p* value	< 4.0	≥ 4.0	*p *value	< 4.0	≥ 4.0
Sex						
Male	0.234	758 (90.9)	76 (9.1)	0.024	502 (57.0)	378 (43.0)
Female		417 (92.3)	35 (7.7)		298 (62.7)	177 (37.3)
icT						
T1/2	0.557	107 (91.5)	10 (8.5)	0.024	88 (67.2)	43 (32.8)
T3/4		1056 (91.3)	101 (8.7)		101 (57.9)	510 (42.1)
icN						
N0	0.453	211 (90.9)	21 (9.1)	0.307	155 (61.5)	97 (38.5)
N1/2		915 (91.4)	86 (8.6)		605 (57.5)	444 (42.3)
RT modality						
3D-CRT	0.48	732 (91.3)	70 (8.7)	< 0.001	532 (62.5)	319 (37.5)
Tomotherapy		443 (91.5)	41 (8.5)		268 (53.2)	236 (46.8)
ypT						
T1/2	0.004	599 (93.6)	42 (6.4)	< 0.001	427 (63.9)	241 (36.1)
T3/4		573 (89.3)	69 (10.7)		371 (54.3)	312 (45.7)
ypN						
N0	0.386	855 (91.5)	79 (8.5)	0.15	574 (58.5)	407 (41.5)
N1/2		318 (90.9)	32 (9.1)		224 (60.2)	148 (39.8)
Adjuvant chemotherapy						
No	0.389	539 (91.0)	53 (9.0)	0.001	343 (54.6)	285 (45.4)
Yes		636 (91.6)	58 (8.4)		457 (62.9)	270 (37.1)
RT fractionation						
LCRT	0.513	1147 (91.3)	109 (8.7)	0.012	776 (58.6)	549 (41.4)
SCRT		28 (93.3)	2 (6.7)		24 (80.0)	6 (20.0)

*3D-CRT* three-dimensional conformal radiotherapy, *LCRT* long-course RT, *SCRT* short-course RT

**Fig. 2:**
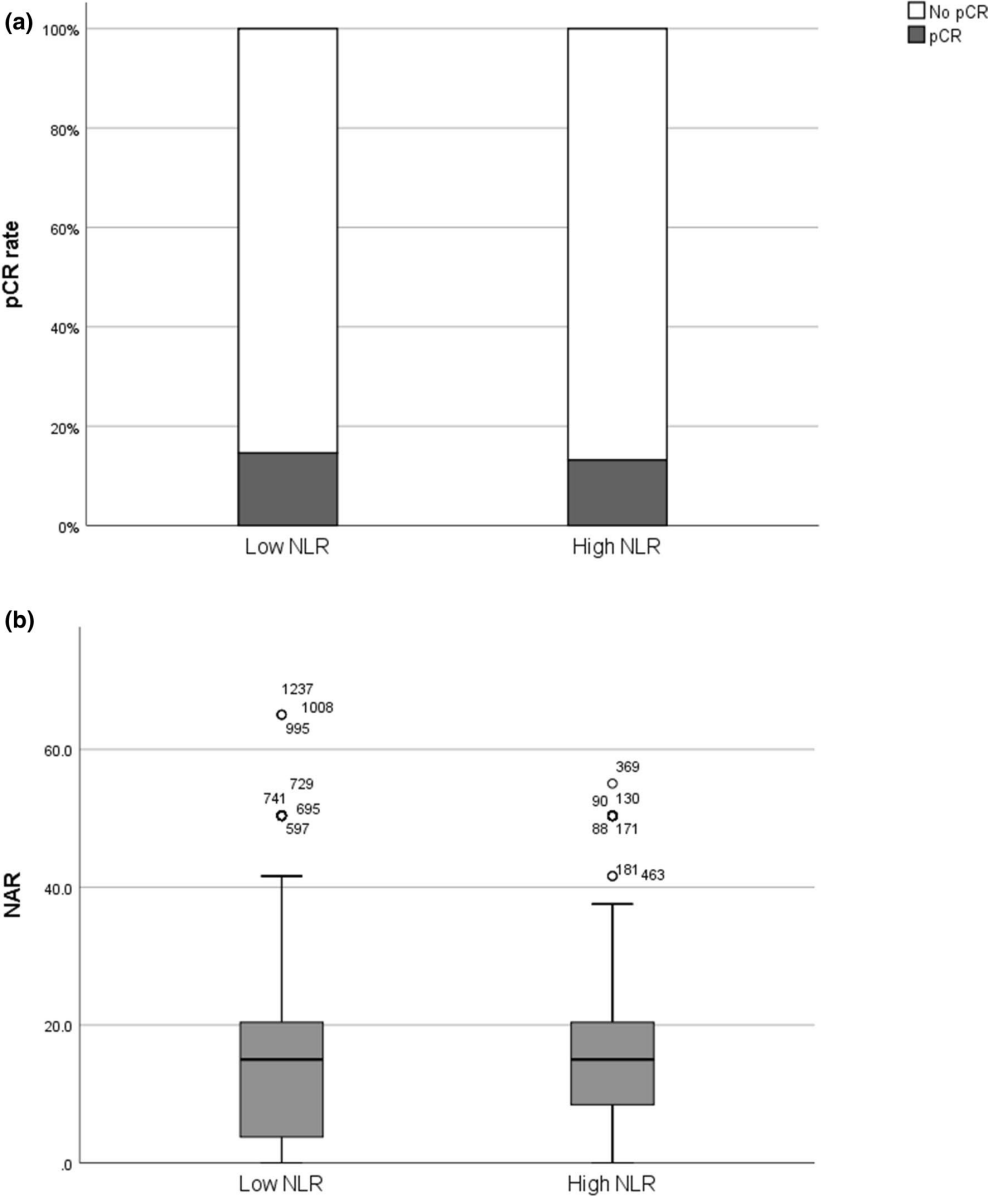
**a** Chi-square test for post-RT NLR and pCR rate. Association between post-radiotherapy (RT) neutrophil-to-lymphocyte ratio (NLR) and the pathologic complete response rate per the chi-square test. **b** Chi-square test for post-RT NLR and NAR. Association between post-RT neutrophil-to-lymphocyte ratio (NLR) and the neoadjuvant rectal score per the chi-square test

**Fig. 3:**
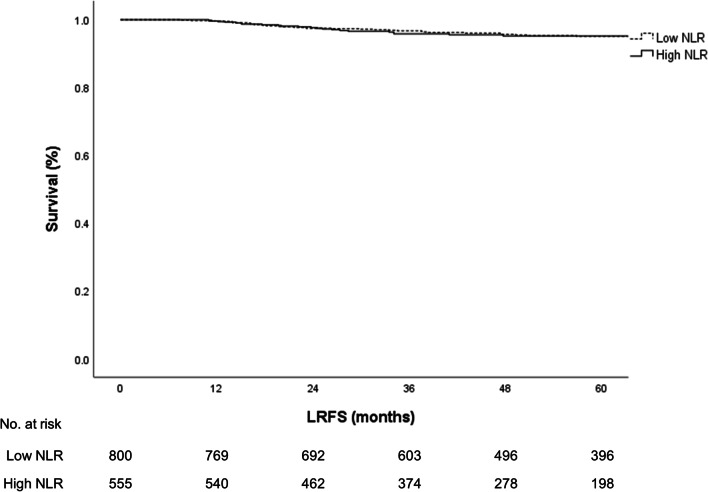
LRFS in patients with low post-RT NLR versus high post-RT NLR. Kaplan–Meier estimates of local recurrence-free survival in patients with low post-radiotherapy (RT) neutrophil-to-lymphocyte ratio (NLR) vs. high post-RT NLR

Following CCRT, pCR was achieved in 187 patients (13.8%). The chi-square test was performed to analyze the association between post-RT NLR and the pCR rate; however, no significant association was found between these variables (*p* > 0.05, Fig. 2a). Moreover, the NAR score and 5-year LRFS were not significantly different between patients with different NLR (*p* > 0.05, Fig. 2b, Fig. 3). In our study, among the 30 patients who were treated with SCRT, 11 patients had low rectal tumors. Among these patients, eight patients (72.7%) achieved downstaging, and one patient (9.1%) achieved pCR. Among the 1325 patients who were treated with long-course radiotherapy, 547 (41.3%) patients had low rectal tumors. Among these patients, 86 patients (15.7%) achieved pCR. Intensification chemotherapy was not administered in our institution. All patients treated with the hypofractionated regimen received 1-week SCRT plus 6-week XELOX (capecitabine 1000 mg/m^2^) and oxaliplatin 130 mg/m^2^ every 3 weeks before TME.

## Discussion

To the best of our knowledge, this is the largest study reporting a relationship between post-treatment leukocytosis and neutrophilia with high DM risk and poor OS among patients with LARC. Increased NLR due to neoadjuvant treatment occurred more frequently in patients with more advanced stages of cancer. In the present study, NLR, but not neutrophil or lymphocyte alone, was significantly associated with an increased risk of development of DM. Furthermore, considering that peripheral blood lymphocytes are sensitive to irradiation, we speculated that RT details may be modifiable factors for NLR, which indirectly affect DMFS [9]. In the era of radiation-induced immunomodulation, preserving the immune status of patients is crucial for improving outcomes [10]. While the pre-treatment immune status is a non-modifiable predictor of progression free survival (PFS), the post-RT immune status is modifiable and was associated with improved PFS in traditional RT cohorts. Using IMRT rather than 3D-CRT, and LCRT rather than SCRT, despite the small sample size of patients who were treated with SCRT, was associated with lymphopenia; however, these parameters are not independently associated with DMFS when accounting for post-RT NLR. Though this hypothesis-generating finding requires further corroboration, it implies that NLR monitoring and the therapeutic manipulation of this parameter can indirectly affect DMFS by attenuating lymphopenia.

A high risk of DM development following TME in patients with LARC was associated with increased NLR following neoadjuvant treatment but not at initial diagnosis in this study. Parallel findings were also reported by investigators from the Mayo clinic [11]. They found that a high NLR pre-treatment (> 3) was significantly associated with worse DFS (HR, 1.36) in all stages for patients with colon, but not rectal, cancer among the 2536 patients with stages I–III colon or rectal cancer. Given that tumor-induced immune suppression is based on an impaired balance between regulatory lymphocytes or abnormal myeloid cells and cytotoxic cells, we can speculate that naïve primary rectal cancer tumor has a weaker correlation with tumor-induced immune suppression than other malignancies. For instance, tumor-derived granulocyte colony-stimulating factor release or kallikrein-related peptidase 5 over-expression in tumor cells was found to be associated with high pre-treatment NLR, which was associated with poor prognosis in patients with uterine cervical cancer [12, 13].

RT and chemotherapy are the leading causes of high NLR in patients with cancer. Radiation-induced lymphopenia is a direct consequence of the irradiation of blood passing through the irradiated body area during RT [14]. Findings on IMRT are interesting [15]. Helical tomotherapy is a dedicated IMRT system that can improve dose conformity at the price of a raised low dose radiation exposure of surrounding tissue. The frequently increased NLR in patients undergoing helical tomotherapy can be explained by the exposure of the bone marrow to low dose radiation dispersed outside the target area [16]. Rose et al. performed multiple studies on patients with cervical cancer undergoing concurrent chemo-RT and a relationship between bone marrow radiation dose–volume metrics and hematologic toxicity and identified hematologically active bone marrow sub-regions based on 18F-fluorodeoxyclucose-positron emission tomography [17, 18]. Therefore, the idea of optimization of active bone marrow sparing-IMRT [19], which was tested and proven in a gynecologic malignancy, is worth considering in LARC as well.

RT fractionation is seemingly critical for NLR evaluation [20]. Conventional fractionation for various solid tumors produced profound lymphopenia and reduced both CD4+ and CD8+ T cells; this effect was sustained for at least 6 months [21]. Reducing the overall RT treatment time seemingly has an important role in the protection against lymphopenia in patients with non-small cell lung cancer [22]. Though SCRT has not been widely adopted in the Republic of Korea or North America owing to concerns regarding toxicity and down-staging effects [2, 23], recent randomized clinical trials suggest incorporating SCRT into the clinical management of patients with LARC as an appealing treatment alternative to LCRT [24]. The randomized Swedish trial, Stockholm III trial, which tested “SCRT followed by delayed surgery,” showed that the downstaging effects of SCRT were similar to those of LCRT [25]. Furthermore, several recent trials, such as the Radiotherapy And Preoperative Induction therapy followed by Dedicated Operation (RAPIDO) and the Short Term radiotherapy followed by chEmotherapy versus Long-term chemoradiotherapy in Locally Advanced Rectal cancer (STELLAR) trial which integrated SCRT into TNT strategy, showed outcomes that were promising or at least comparable with those of standard LCRT [24, 26]. These studies demonstrated that when the time interval between SCRT and surgery is delayed, SCRT seems to have outcomes comparable with LCRT. In this study, DM risk seemingly increased in patients with elevated NLR following treatment. TNT has been more commonly used than other treatment methods in patients with LARC owing to its several theoretical advantages [27]. Considering that the data in the literature are insufficient to consider TNT as a standard treatment, efforts should be made to select the most appropriate candidates for TNT. NLR during the treatment course can be a potential marker in addition to other biomarkers, such as RAS and/or RAF mutations, microsatellite instability, and magnetic resonance imaging-based radiomics signature, to select patients for more intensified or individualized treatments, such as TNT [28, 29].

The theoretical benefit of TNT regarding DM has been realized in the Partenariat de Recherche en Oncologie Digestive (PRODIGE) 23 (3-year HR, 0.64; and 95% CI 0.44–0.93) and RAPIDO (3-year HR, 0.69; and 95% CI 0.53–0.89) trials [24, 30]. In PRODIGE 23, FOLFIRNOX was used in the TNT arm; thus, the improved outcomes of patients in the TNT arm may be partly owing to the addition of irinotecan. The experimental arm in the RAPIDO trial received SCRT followed by consolidative CapeOx or FOLFOX, and TME. The authors speculated that the reduction in DM might be owing to the better compliance to preoperative chemotherapy than to adjuvant chemotherapy when offered in the standard treatment group. Considering that lymphocyte counts and NLR status were associated with more frequent abscopal (out-of-RT field) responses in patients with advanced cancer who underwent combined immunotherapy and RT [31], we speculated that adding SCRT to TNT may play a role in DM control by mitigating increased NLR risk. RT upregulates regulatory T cells, myeloid-derived suppressor cells, and M2 tumor-associated macrophages; these cells promote an immunosuppressive milieu, and identifying and addressing these unforeseen variables in addition to NLR may enable more robust DM control than addressing only NLR [32].

The following are several other ways to improve outcomes by modulating NLR: Proton or carbon ion RT seemingly produces much less lymphopenia and less increases in NLR than conventional RT [33]. Despite conflicting results, some studies suggest a strong effect of amifostine (WR2721) on protecting lymphocytes against radiation [34]. Byun et al. reported that the combination treatment of interleukin-7 and RT not only recovered RT-induced lymphopenia but also suppressed tumor growth in a mouse model [35]. The selective depletion of immunosuppressive cells, such as regulatory T cells or myeloid-derived suppressor cells, is another exciting approach [6].

In our study, LRFS, pCR rate, and NAR scores were not significantly different between patients with different NLRs, and this is consistent with other studies [11, 36]. It is possible that, owing to the small number of events related to local recurrence or the pCR rate in our cohort, this study did not have the power to detect the impact of NLR on these outcomes in patients with LARC. In contrast, we speculated that different radio-resistances of tissue-resident T cells [37], which are more radio-resistant than circulating T cells, could explain these findings as the presence and function of tumor-infiltrating lymphocytes in tumor cells seemingly promotes regression and a high pCR rate. However, further studies in this regard are warranted.

This study has some limitations. The current study is retrospective and has a long-time frame (13 years), implying that it is subject to selection bias, though we attempted to apply as few exclusion criteria as possible. The year of diagnosis that was included in the analysis had an impact on the OS, but not on the risk of DMFS (Table 2), which means that the potential time-dependent confounders would not obscure our main findings. Hematologic parameters are often influenced by numerous other uncontrollable factors; therefore, potential confounding can never be excluded. We attempted to confirm whether there were other reasons for increased NLR such as infection, systemic inflammatory response (SIR), or sepsis, and reviewed the medical records to examine if there were any patients who experienced SIR or sepsis. Although no patient experienced infection or sepsis or was treated with antibiotics owing to infection during the course of or within 3 months after CCRT, there might be other unforeseen confounding factors. Using antibiotics or corticosteroids may influence the results of this study. The cut-off value for post-RT NLR in our study may not be the ideal cut-off value, and further validation is required to determine the optimal cut-off value for NLR. The small number of patients who underwent SCRT and use of XELOX chemotherapy for 6 weeks before TME in our ongoing pilot single arm prospective study (NCT03676517) can obscure the results of data analyses. Nevertheless, these shortcomings do not diminish the potential of our findings or the clinical implications of RT in patients with LARC.

## Conclusions

In patients with LARC, increased NLR during neoadjuvant treatment, not before treatment, is associated with an increased risk of development of DM and poor OS without affecting pathologic primary tumor regression and local control. Increasing NLR during treatment is directly related to RT fractionation and delivery modality, and tumor characteristics. Therefore, combining SCRT with 3D-CRT or active bone marrow sparing-IMRT may be modifiable factors that can indirectly affect DMFS by attenuating NLR in patients with LARC. These results are hypothesis generating only, and confirmatory studies are required.

## Supplementary Information

**Additional file 1.** The optimal cut-off value for post-RT NLR.

**Additional file 2.** The Kaplan–Meier curve of the probability of NLR reaching its peak.

## Data Availability

Research data are stored in an institutional repository and will be shared upon request to the corresponding author.

## Abbreviations

CI: Confidence interval
DFS: Disease-free survival
DM: Distant metastasis
IMRT: Intensity-modulated radiation therapy
LARC: Locally advanced rectal cancer
LRFS: Local recurrence-free survival
OR: Odds ratio
NLR: Neutrophil-to-lymphocyte ratio
PFS: Progression free survival
RT: Radiotherapy
TME: Total mesorectal excision
TNT: Total neoadjuvant therapy
3D-CRT: Three-dimensional conformal radiotherapy
