# Supplementary material for: Association of neutrophil-to-lymphocyte ratio, radiotherapy fractionation/technique, and risk of development of distant metastasis among patients with locally advanced rectal cancer
Source: Radiat Oncol. 2022 May 21;17:100. doi: 10.1186/s13014-022-02065-8 (PMC9123758; doi:10.1186/s13014-022-02065-8)
Supplement: Supplementary file 1 — Additional file 1. The optimal cut-off value for post-RT NLR. [file 13014_2022_2065_MOESM1_ESM.docx]

**Additional file 1. The optimal cut-off value for post-RT NLR**

|  | **HR** | **95% CI** | **p value** |
| --- | --- | --- | --- |
| Pre-RT NLR | |  |  |
| ≥2.0 vs.＜2.0 | 1.14 | 0.89-1.4719 | 0.304 |
| ≥2.5 vs.＜2.5 | 1.12 | 0.86-1.44 | 0.404 |
| ≥3.0 vs.＜3.0 | 1.08 | 0.80-1.45 | 0.618 |
| ≥3.5 vs.＜3.5 | 1.08 | 0.76-1.53 | 0.676 |
| ≥4.0 vs.＜4.0 | 1.25 | 0.83-1.88 | 0.789 |
|  |  |  |  |
| Post-RT NLR | |  |  |
| ≥3.0 vs.＜3.0 | 1.2 | 0.96-1.51 | 0.113 |
| ≥4.0 vs.＜4.0 | 1.44 | 1.14-1.80 | 0.002 |
| ≥5.0 vs.＜5.0 | 1.44 | 1.14-1.81 | 0.003 |
| ≥6.0 vs.＜6.0 | 0.04 | 1.02-1.66 | 0.035 |
| ≥7.0 vs.＜7.0 | 1.16 | 0.89-1.51 | 0.275 |

NLR: neutrophil-to-lymphocyte ratio; CI: confidence interval
