# Supplementary figures and images for: Association of neutrophil-to-lymphocyte ratio, radiotherapy fractionation/technique, and risk of development of distant metastasis among patients with locally advanced rectal cancer
Source: Radiat Oncol. 2022 May 21;17:100. doi: 10.1186/s13014-022-02065-8 (PMC9123758; doi:10.1186/s13014-022-02065-8)

**Additional file 2. The Kaplan-Meier curve of the probability of NLR reaching its peak**


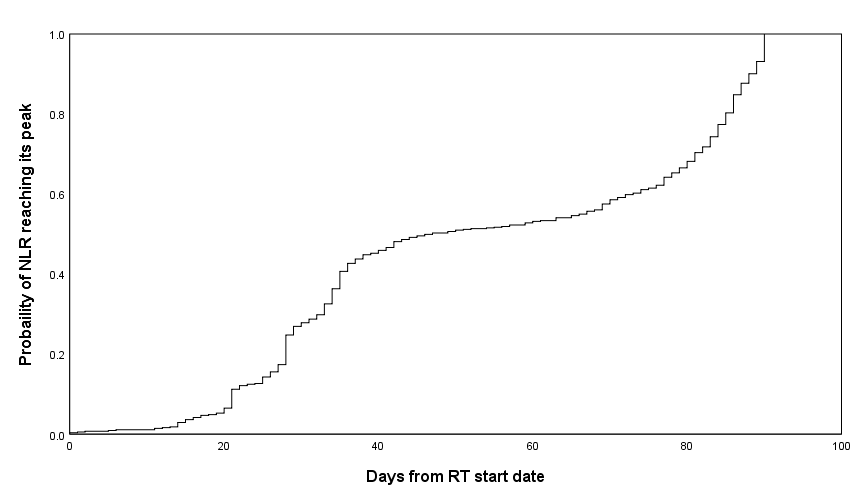

Supplement: Supplementary file 2 — Additional file 2. The Kaplan–Meier curve of the probability of NLR reaching its peak. [file 13014_2022_2065_MOESM2_ESM.docx]
